# Supplementary material for: Cardiopulmonary Arrest and Resuscitation in the Prone Patient: An Adult Simulation Case for Internal Medicine Residents
Source: MedEdPORTAL. 2021 Feb 11;17:11081. doi: 10.15766/mep_2374-8265.11081 (PMC7880259; doi:10.15766/mep_2374-8265.11081)
Supplement: Supplementary file 1 — Simulation Case Template.docxLearner Information.docxDebriefing Materials.docxProne CPR Operating Procedure.docx [file mep_2374-8265.11081-s001.zip › A. Simulation Case Template.docx]

| **Appendix A: MedEdPORTAL Simulation Case Template**  **SIMULATION CASE TITLE:** *Cardiopulmonary Arrest and Resuscitation in the Proned Patient : An Adult Simulation Case for Internal Medicine Residents*  **AUTHORS: Tejas Sinha, MD, Kyle Stinehart, MD, Cashay Moorer, MPH, Carleen Spitzer, MD**  **LEARNER AUDIENCE: Internal Medicine Residents** | |
| --- | --- |
| **PATIENT NAME: Robert Ware**  **PATIENT AGE: 70**  **CHIEF COMPLAINT: Fevers and Shortness of Breath**  **PHYSICAL SETTING: Intensive Care Unit** | |
|  | |
| **Brief narrative description of case** | *Include the presenting patient chief complaint and overall learner goals for this case*  The patient is a 70 year old man with a past medical history of Diabetes Mellitus, Hypertension who presented with fevers and shortness of breath one week ago and was diagnosed with influenza. He became progressively more hypoxic and was transferred to the intensive care unit with concerns for acute respiratory distress syndrome two days ago. Given his hypoxia, he was proned two hours ago. A code blue is called for a cardiac arrest*.* |
| **Primary Learning Objectives** | By the end of the case, the learners will be able to:  1. Identify PEA arrest and initiate CPR in a prone patient with the appropriate ACLS algorithm  2. Demonstrate when to turn a prone patient to the supine position during a cardiac arrest  3. Evaluate potential causes of a PEA arrest; recognize and treat tension pneumothorax  4. Demonstrate effective teamwork in caring for a critically ill patient  5. Organize a team “debriefing” following the code event |
| **Critical Actions** | -Identify PEA arrest and start chest compressions in a prone patient  -Place patient on defibrillator and follow the ACLS algorithm  -Decide when to safely turn patient supine while ensuring safety of endotracheal tube and associated lines (i.e. central venous catheter and arterial line)  -Identify tension pneumothorax as cause for PEA arrest  -Attempt needle decompression to resolve tension pneumothorax  -Practice formal debriefing process after code blue  -Identify need for chest tube placement after needle decompression  -Evaluate appropriateness for targeted temperature management |
| **Learner Preparation or Prework** | Learners were given information about our institutions Prone CPR standard operating procedure at the start of the academic year prior to conducting the simulation |

| Initial Presentation | | | |
| --- | --- | --- | --- |
| **Initial vital signs** | Temp-102.1, HR-130, BP: 0/0 (PEA Arrest), SpO2: unavailable on FiO2-100% | | |
| **Overall Setting and Appearance** | Learners are called into the room with the sound of a code blue alarm. They are informed that they are in the intensive care unit. When they enter, the mannequin will be intubated and proned with the mechanical ventilator alarming. | | |
| **Confederates (e.g., standardized participants) and their roles in the room at case start** | No one is present in the room at the beginning of the case. The team is informed by the simulationist over the speaker that the patient just lost a pulse and that the ventilator was alarming prior to the code. | | |
| **HPI** | Written information about the details of the case as detailed in Appendix B is provided prior to the case. | | |
| **Past Medical/Surgical History** | **Medications** | **Allergies** | **Family History** |
| Diabetes Mellitus  Hypertension | Aspirin  Lisinopril  Amlodipine  Carvedilol  Glargine | Penicillin | HTN and Lung Cancer in Father-Deceased  Mother-Unknown |
| **Physical Examination** | | | |
| **General** | intubated, sedated, prone position | | |
| **HEENT** | sedated, endotracheal tube 23 cm at lip, pupils equal and reactive | | |
| **Neck** | right internal jugular CVC in place, no carotid bruit bilaterally | | |
| **Lungs** | decreased breath sounds over right hemithorax, coarse breath sounds over left hemithorax | | |
| **Cardiovascular** | absent heart sounds | | |
| **Abdomen** | soft, non-distended | | |
| **Neurological** | sedated, unresponsive to painful stimuli | | |
| **Skin** | unremarkable | | |
| **GU** | unremarkable | | |
| **Psychiatric** | sedated, unresponsive | | |

| **Mannequin Details** | |
| --- | --- |
| Head: intubated, CVC in right IJ, endotracheal tube in place connected to mechanical ventilator | Legs/Feet: NA |
| Chest: telemetry leads | Arms/Hands: 20g IV, left arterial line |
| Abdomen: NA | Pelvic: NA |
| Back: NA |  |

| Instructor Notes - Changes and CASE Branch Points | | |
| --- | --- | --- |
| **Intervention / Time point** | **Change in Case** | **Additional Information** |
| Start of Case | Code Blue is called | Informed by RN that ventilator has been alarming with elevated peak and plateau pressures prior to loss of pulse. |
| CPR Initiated | Decision to turn patient supine | Facilitator will evaluate quality of prone compressions, monitor/defibrillator attachment, and use of counter-pressure with a sand bag or rigid back board |
| Patient is flipped supine or prompted to do so by facilitator after 4 minutes | Persistent PEA after being flipped supine with elevated peak and plateau pressures | Ensure that safety of tubes and line is prioritized when patient is turned supine |
| Team verbalizes reversible causes of PEA Arrest (H’s & T’s) |  |  |
| Team fails to identify tension pneumothorax as etiology of cardiac arrest | Patient remains in persistent PEA arrest until case concludes after 15 minutes of simulation time |  |
| Team identifies tension pneumothorax as likely etiology of cardiac arrest and attempts needle decompression | Return of spontaneous circulation |  |
| Return of Spontaneous circulation | End of Case | Team prompted by facilitator playing ICU fellow to consider placement of chest tube and targeted temperature management if not already verbalized by the team |
|  |  |  |
|  |  |  |

**Ideal Scenario Flow**

The learners will enter the room to find a prone patient without a pulse with the ventilator alarming due to high peak pressures. If/when residents ask, they are informed that the plateau pressures have also been high. They will use effective team work and communication skills to fulfill the various roles needed for effective management of a cardiac arrest as per ACLS (Advanced Cardiovascular Life Support). They will begin two-handed compressions on the back between the lower ends of the scapulae over the thoracic spine with use of counter pressure with either a rigid backboard or a sandbag under the lower half of the sternum. The monitor will be attached with placement of the defibrillator pads on the left upper back and left lateral chest. The team will perform a pulse and rhythm check and identify PEA as the type of cardiac arrest. The code leader will decide when to turn the patient supine (seeking input from other teammates as appropriate). Ideally this should occur during a pulse and rhythm check to avoid disrupting chest compressions. He or she should ensure that there is adequate communication so that when the patient is turned supine all tubes and lines are accounted for and protected. Once supine, the team will resume CPR and discuss the reversible causes of a PEA cardiac arrest. They will recognize tension pneumothorax as the most likely cause of the arrest in a patient with decreased breath sounds over the right hemithorax, severe ARDS and acutely elevated peak and plateau pressures on the ventilator. They will attempt needle decompression which will lead to return of spontaneous circulation. The case will conclude with the team verbalizing the need for a chest tube and a discussion of the utility of targeted temperature management.

**Anticipated Management Mistakes**

1. Difficulty with CPR in a prone patient: We found that our learners were largely unfamiliar with the standard operating procedure for prone CPR. Many did not know where to place hands for compressions and were unfamiliar with the use of a sandbag or rigid back board for counter-pressure. The standard operating procedure was reviewed during the debrief.
2. Difficulty with determining when to turn patient supine: Many of our learners were unfamiliar with when to turn the patient supine. Some teams turned the patient supine before starting compressions or determining the underlying rhythm. This decision was reviewed in the debriefing, and importance of minimizing interruptions to chest compressions was reinforced.
3. Difficulty with interpretation of elevated peak and plateau pressures: A few teams did not recognize that the elevated peak and plateau pressures could be suggestive of an underlying tension pneumothorax. The definitions of peak and plateau pressures was reviewed during the debrief as well as common causes for elevations in each measurement.
4. Difficulty with bedside monitors: Some of our learners struggled with correctly attaching the defibrillator pads in a prone patient. The standard operating procedure was reviewed and it was reiterated that pads should be placed left upper back and left lateral chest
