# Supplementary material for: Cardiopulmonary Arrest and Resuscitation in the Prone Patient: An Adult Simulation Case for Internal Medicine Residents
Source: MedEdPORTAL. 2021 Feb 11;17:11081. doi: 10.15766/mep_2374-8265.11081 (PMC7880259; doi:10.15766/mep_2374-8265.11081)
Supplement: Supplementary file 1 — Simulation Case Template.docxLearner Information.docxDebriefing Materials.docxProne CPR Operating Procedure.docx [file mep_2374-8265.11081-s001.zip › B. Learner Information.docx]

*****[**Scenario Intro (**read to learner**)**](#Instructions)

The patient is a 70 year old man with a past medical history of DM, HTN who presented with fevers and shortness of breath one week ago and was diagnosed with influenza. He became progressively more hypoxic and was transferred to the MICU with concerns for ARDS two days ago. Given his hypoxia, he was proned two hours ago. A Code Blue is called for a PEA arrest.

| Name: Robert A. Ware | Date of Birth: 2/17/1950 | Age: 67 |
| --- | --- | --- |
| Height: 5’11’’ | **Weight:** 87 kg | **MRN:** XXXXXXXXX |

| Patient History | |
| --- | --- |
| Past Medical: DM, HTN | **Past Surgical:** Left knee replacement |
| Medications:  ASA, glargine, lisinopril, amlodipine, carvedilol | **Allergies:** Penicillin (rash) |

| Review Of Systems | | |
| --- | --- | --- |
| CNS: N/A | **Renal:** N/A | |
| Cardiovascular: N/A | **Abdominal:** N/A | |
| Pulmonary: N/A | **Psychiatric:** N/A | |
| Physical Exam | | |
| Head: NC/AT. ETT in place. PERRL | | **Cardiac:** PEA |
| Chest (Initial exam): decreased breath sounds over right hemithorax; coarse breath sounds over left hemithorax | | **Legs:** No edema |
| Abdomen: Unable to assess – patient proned | | **Back:** No rash |

| History of Present Illness | |
| --- | --- |
| Events Leading up to Illness: Patient was admitted for progressive hypoxia secondary to the flu. He is now intubated/sedated for ARDS and has been proned because his P/F ratio is now < 150. Current vent settings are AC/VC: Vt 6cc/kg; RR 24; PEEP 14; FiO2 70%. | |
| Level of Consciousness (LOC): Sedated immediately prior to arrest | **Vitals on Admission:**  Temp 102.1; BP 110/68; HR 110 |
| Onset of Symptoms: 7 days | **Severity of Pain:** Unable to assess |
| Provocation/Provokes: N/A | **Current Interventions:** Intubated. Now also receiving broad-spectrum antibiotics and oseltamivir. |

| Labs/Images/Testing | | | | | | | |
| --- | --- | --- | --- | --- | --- | --- | --- |
| Test | Results: | | | | | | |
| CBC | WBC: 13.0 | RBC: | Hbg: 11.5 | Hct: 34 | Platelets: 151 |  |  |
| Chem 7 | Na: 132 | K: 4.2 | Cl: 109 | C02: 20 | Glu: 169 | BUN: 31 | Creatinine: 1.4 |
| ABG | pH: 7.15 | PaC02: 68 | Pa02: 56 | Hb03: 20 | Sa02: 89 |  |  |
| Troponin |  | | | | | | |
| CT |  | | | | | | |
| X-Ray |  | | | | | | |
| Ultrasound |  | | | | | | |
| Other |  |  |  |  |  |  |  |
