# Supplementary material for: Cardiopulmonary Arrest and Resuscitation in the Prone Patient: An Adult Simulation Case for Internal Medicine Residents
Source: MedEdPORTAL. 2021 Feb 11;17:11081. doi: 10.15766/mep_2374-8265.11081 (PMC7880259; doi:10.15766/mep_2374-8265.11081)
Supplement: Supplementary file 1 — Simulation Case Template.docxLearner Information.docxDebriefing Materials.docxProne CPR Operating Procedure.docx [file mep_2374-8265.11081-s001.zip › C. Debriefing Materials.docx]

**Debrief Framework**

- Ask learners open ended questions about the case,
  - What went well?
  - What could have been done better?
- Review actions while patient was prone
  - Was prone ACLS initiated and executed appropriately
    - Emphasize critical actions and technique from learning points below
- Discuss how the decision to turn the patient supine was made and review the execution
- Review performance of ACLS, recognition of tension pneumothorax as the cause for the arrest and immediate need for needle decompression
- Discuss post cardiac arrest care including consideration of targeted temperature management and need for chest tube placement.

**Prone CPR Case Learning Points**

- Appropriate ACLS management of PEA arrest in a prone patient
  - Discuss potential causes of PEA arrest (H’s & T’s)
    - Recognize tension pneumothorax as cause of arrest and appropriately needle decompress
  - Demonstrate appropriate compressions of patient in prone position
    - Two hands between lower ends of scapulae over thoracic spine
    - Insert rigid backboard under patient
    - If able, insert sand bag under the lower half of the sternum to provide counter pressure
    - Place defibrillator pads on left upper back and left lateral chest
    - Remind learners that if additional IV access needed, place humeral IO
  - Prioritize turning patient supine
    - Should be clear communication to all members of code team when decision is made to turn patient supine
    - Ensure adequate safety of all tubes and lines

**Flow Diagram for CPR in the Prone Position**

Yes

Patient noted to be pulseless/unresponsive while in the prone position

Start CPR in the prone position; place backboard, sand bag; attach pads

Are there enough people available to flip the patient supine (minimum 5-6 people required; may require more pending size/habitus of patient)?

Develop a plan to flip the patient supine (direct responsibility assigned for airway, lines, tubes, etc)

Continue CPR/ACLS in the prone position

No

Once plan is set/verbalized, plan to supine patient at next 2 min pause for pulse check

Complete first round of ACLS
