# Supplementary material for: Cardiopulmonary Arrest and Resuscitation in the Prone Patient: An Adult Simulation Case for Internal Medicine Residents
Source: MedEdPORTAL. 2021 Feb 11;17:11081. doi: 10.15766/mep_2374-8265.11081 (PMC7880259; doi:10.15766/mep_2374-8265.11081)
Supplement: Supplementary file 1 — Simulation Case Template.docxLearner Information.docxDebriefing Materials.docxProne CPR Operating Procedure.docx [file mep_2374-8265.11081-s001.zip › D. Prone CPR Operating Procedure.docx]

Ohio State Wexner Medical Center: Standard Operating Procedure: CPR in Prone Patients

1. Process Details
2. Cardiac arrest is diagnosed in a patient being ventilated in the prone position
3. Assemble code team per OSUWMC guidelines and standard procedures
4. Immediately begin two-handed compressions on the back
   1. Compressor body and hand positioning at the bedside is similar to standard CPR
   2. Compressor hands should be placed in between the lower ends of the scapulae over the thoracic spine (corresponding to approximately the thoracic vertebrae numbers 7-10)
5. Insert a rigid back board under the patient
6. If able, insert a sandbag under the lower half of the sternum to provide counter-pressure
7. Place defibrillator pads on patient on the left upper back and left lateral chest, as shown in figure 2
8. Continue CPR per standard ACLS protocol
   1. Monitor CPR quality with ETCO2 or aterial line, if available
9. If additional IV access is needed while the patient is prone, placement of a humeral IO device is recommended
10. Prepare to turn the patient to supine if/when feasible and safe from a CPR and resuscitation standpoint
    1. Turning the patient supine should occur during a pulse and rhythm check to avoid interruption in high quality chest compressions
    2. Decision to turn the patient supine belongs to the code leader with input from the rest of the members of the code team
    3. All code team members should be made aware of plan to turn the patient supine
    4. Team members should identify themselves as responsible for ensuring the safety of tubes and lines during the turning process
11. Defibrillator pads can be left in place as is or repositioned anteriorly, as circumstances dictate
12. Continue CPR in the supine position
