# Supplementary material for: Cardiopulmonary Arrest and Resuscitation in the Prone Patient: An Adult Simulation Case for Internal Medicine Residents
Source: MedEdPORTAL. 2021 Feb 11;17:11081. doi: 10.15766/mep_2374-8265.11081 (PMC7880259; doi:10.15766/mep_2374-8265.11081)
Supplement: Supplementary file 1 — Simulation Case Template.docxLearner Information.docxDebriefing Materials.docxProne CPR Operating Procedure.docx [file mep_2374-8265.11081-s001.zip › _Educational_Summary_Report_11081.pdf]

MedEdPORTAL® The AAMC Journal of Teaching and Learning Resources

Original Publication Open Access

Cardiopulmonary Arrest and Resuscitation in the Prone Patient: An Adult Simulation Case for Internal Medicine Residents

Tejas Sinha, MD*, Kyle Stinehart, MD, Cashay Moorer, MPH, Carleen Spitzer, MD

*Corresponding author: tsinha24@gmail.com

Abstract

Introduction: Acute respiratory distress syndrome (ARDS) is present in approximately 10% of ICU admissions and is associated with great morbidity and mortality. Prone ventilation has been shown to improve refractory hypoxemia and mortality in patients with ARDS. **Methods:** In this simulation, a 70-year-old male had been transferred to the ICU for ARDS and was undergoing scheduled prone ventilation as part of his care when he experienced a cardiopulmonary arrest secondary to a tension pneumothorax. Learners demonstrated how to manage cardiac arrest in a prone patient and subsequently identified and treated the tension pneumothorax that was the cause of his initial arrest. This single-session simulation for internal medicine residents (PGY 1-PGY 4) utilized a prone mannequin connected to a ventilator in a high-fidelity simulation center. Following the simulation, facilitators led a team debriefing and reviewed key learning objectives. **Results:** A total of 103 internal medicine residents participated in this simulation. Of those, 43 responded to a postsimulation survey. Forty-two of 43 agreed or strongly agreed that all learning objectives were met, that the simulation was appropriate for their level of training, and that their participation would be useful for their future practice. **Discussion:** We designed this simulation to improve learners' familiarity with prone cardiopulmonary resuscitation and to enhance overall comfort with cardiac arrest management. Postsimulation survey results and debriefings revealed that the simulation was a valuable education opportunity, and learners felt that their participation in this simulation would be helpful in their future practice.

Keywords
Critical Care, ARDS, Prone CPR, Code Blue, Hypoxia, Critical Care Medicine, Internal Medicine, Pulmonary Medicine, Simulation

Educational Objectives

By the end of this activity, learners will be able to:

1. Identify pulseless electrical activity (PEA) arrest and initiate CPR in a prone patient with the appropriate advanced cardiovascular life support algorithm.
2. Demonstrate when to turn a prone patient to the supine position during a cardiac arrest.
3. Evaluate potential causes of a PEA arrest and recognize and treat tension pneumothorax.
4. Demonstrate effective teamwork in caring for a critically ill patient.
5. Organize a team debriefing following the code event.

Citation:
Sinha T, Stinehart K, Moorer C, Spitzer C. Cardiopulmonary arrest and resuscitation in the prone patient: an adult simulation case for internal medicine residents. *MedEdPORTAL*. 2021;17:11081.
https://doi.org/10.15766/mep_2374-8265.11081

Introduction

Acute respiratory distress syndrome (ARDS) is a type of acute, diffuse, inflammatory lung injury characterized by noncardiogenic pulmonary edema, poor lung compliance, increased physiologic dead space, and severe hypoxia despite positive pressure ventilation.[1,2] ARDS is often underrecognized and has a prevalence of greater than 10% of admissions to the ICU.[3] This condition has great morbidity and mortality associated with it,[4] and so, a significant amount of research has been dedicated to identifying strategies that may improve patient outcomes. To date, prone ventilation is one of the most widely accepted strategies for improving outcomes in patients with ARDS,[5-9] and given the current COVID-19 pandemic, its use is expected to increase.[10,11]

Given the high morbidity and mortality associated with ARDS and the recent increase in patients suffering from acute hypoxemic respiratory failure/ARDS who are undergoing prone ventilation, it is of utmost importance that clinicians understand how to perform CPR in the prone position. CPR in the prone position

Copyright © 2021 Sinha et al. This is an open-access publication distributed under the terms of the Creative Commons Attribution license.

has been shown to be effective,[12] and current American College of Cardiology/American Heart Association recommendations state that it “may be reasonable for rescuers to provide CPR with the patient in the prone position, particularly in hospitalized patients with an advanced airway,” when the patient cannot be placed in the supine position.[13] Despite this, most residents are unfamiliar with prone CPR. For example, in a simulation study of anesthesia residents managing cardiac arrest due to ventricular fibrillation in a prone pediatric patient, investigators found that all of the residents needed additional training in recognizing the rhythm and starting chest compressions in a prone patient.[14] Though we were able to identify one simulation case centered around the use of extra corporeal membrane oxygenation and ARDS,[15] as well as multiple others on the management of tension pneumothorax,[16-20] there is a paucity of simulation literature concerning utilization of prone ventilation in ARDS.

The high incidence and complexity of ARDS care and lack of comprehensive educational modalities on management led us to develop this simulation case for residents of all years in our training program. To our knowledge, this case represents the first simulation designed for trainees to practice resuscitation in a prone patient with ARDS.

Methods

Development

Our institution developed and disseminated a standard operating procedure for CPR in prone patients at the beginning of the academic year.[21] The standard operating procedure emphasized the principles of prone CPR including placement of hands for compressions, use of a rigid surface for counterpressure, placement and use of defibrillation pads in the prone position, and knowledge of when it is safe or necessary to prioritize turning the patient supine. Facilitators evaluated the application of these principles as well as teamwork and overall management of a pulseless electrical activity (PEA) cardiac arrest.

Equipment/Environment

To run the simulation, we utilized a SimMan 3G mannequin in a high-fidelity simulation center. Prior to beginning the case, we connected the mannequin to a mechanical ventilator in the prone position (see Appendix A). A crash cart with standard advanced cardiovascular life support (ACLS) equipment and medication vials was in the room with a defibrillator. Of note, if residents requested a portable chest radiograph, preceptors informed them that one was not immediately available in order encourage them to rely on their physical exam skills and clinical decision-making. For centers that do not have access to a ventilator in their simulation center, facilitators could merely tell participants to assume that the mannequin is attached to a ventilator that is alarming for persistently high peak pressures.

Personnel

One facilitator and one simulation specialist conducted each simulation event. The simulation specialist was responsible for adjusting the monitors and providing additional information as requested by the participants, while the facilitator aided with directing the progression of the scenario and voicing the roles of the nurse and pulmonary fellow, as appropriate. Our facilitators included internal medicine chief residents, a hospitalist, and a pulmonary and critical care fellow and attending.

Implementation

We implemented this 30-minute simulation on an academic half-day with six groups of internal medicine residents. The simulation began with a 5-minute setup of the mannequin and simulation room, as detailed in Appendix A. We provided an introductory briefing including a scenario introduction, history and physical exam findings, and laboratory values for learners, as detailed in Appendix B. There were approximately six to seven residents in each simulation group. We started the case with a code being announced as the learners entered the simulation room. In the room, learners found a pulseless, unresponsive mannequin in the prone position connected to a ventilator. The ventilator was alarming due to high peak pressures. Facilitators evaluated the trainees on their ability to perform CPR in the prone position correctly, their ability to connect the mannequin to a defibrillator, and their decision regarding when to turn the mannequin supine. After the learners turned the mannequin supine, facilitators evaluated their ability to discuss the differential for a PEA arrest and recognize tension pneumothorax as the underlying etiology of this particular code event. Facilitators concluded the case after needle decompression of the pneumothorax, and learners verbalized their plans for postarrest management, including placement of a chest tube and consideration of targeted temperature management. After the conclusion of the case, facilitators spent 10 minutes with the trainees debriefing the simulation. During the debriefing session, facilitators reviewed key learning points for the case (Appendix C), including our institution’s standard operating procedure for prone CPR (Appendix D). At the end of the academic half-day, one of the internal medicine chief residents sent an email to all simulation participants with a link and QR codes to anonymously complete a postsimulation survey.

Assessment

We evaluated trainees on their ability to complete critical actions at various points in the simulated patient’s clinical course. First,

Copyright © 2021 Sinha et al. This is an open-access publication distributed under the terms of the Creative Commons Attribution license.

while the mannequin was prone, we evaluated trainees' ability to identify a PEA cardiac arrest, start compressions, and connect the prone patient to a defibrillator. We then evaluated their decision regarding when to flip the patient to the supine position as well as their execution of the maneuver—noting whether or not the safety of lines and tubes was prioritized. Once the mannequin was supine, we evaluated the trainees' adherence to ACLS metrics and their ability to identify tension pneumothorax as the cause of the arrest. Finally, after needle decompression of the pneumothorax with return of spontaneous circulation, we assessed trainees' ability to recognize the need for chest tube placement and consideration of targeted temperature management.

Debriefing

To allow for learner self-evaluation, facilitators conducted the debriefing in the gather-analyze-summarize format. First, to start the debriefing process, facilitators asked trainees open-ended questions including "What do you think went well?" and "What do you think could have gone better?" After learners had the opportunity to reflect on their own performance, facilitators provided feedback with observations on both correct and incorrect steps. Finally, the critical actions and learning objectives of the simulation were summarized using the facilitator's key learning points and our institution's prone CPR standard operating procedure (Appendices C and D, respectively).

Results

We ran this simulation scenario simultaneously in two adjacent simulation labs, with groups of six to seven residents, three times on three separate dates. A total of 103 internal medicine residents ranging from PGY 1 to PGY 4 completed the case. After the session, 42% of residents completed a postsimulation survey. This survey evaluated learner reactions to the case as well as perceived knowledge gained. Educational objectives were included in the postsimulation survey for reference. Ninety-five percent of residents strongly agreed or agreed that the stated objectives were met. When asked if the simulation scenario was appropriate for their level of training, 98% of respondents strongly agreed or agreed. Similarly, 98% strongly agreed or agreed when asked if the case was useful for future clinical practice.

Discussion

ARDS is a devastating illness that has great morbidity and mortality. Given the data suggesting a mortality benefit with prone ventilation, our medical ICU leaders have emphasized the importance of early prone ventilation in patients with ARDS over the past several years. As part of this initiative, our institution created a standard operating procedure to address how to perform CPR in the prone position. We developed this simulation to disseminate the information in our institution's prone CPR standard operating procedure and to help our residents better apply the information within a deliberate and reflective practice framework. Though we developed this case in the summer of 2019, it is even more relevant now given the ongoing COVID-19 pandemic than it was when we first completed it. Over the course of the past several months, it is now increasingly likely that cardiopulmonary arrests will occur in the prone position,[22] and there has been a call for more evaluation, education, and guidance when completing CPR in the prone position in patients with COVID-19.[22-24] Though our simulation does not involve a COVID-positive patient, we believe that it provides a basic framework upon which to build when considering prone CPR in any patient.

Beyond understanding how to effectively perform CPR and defibrillation in the prone position, it is critical for all internal medicine residents to follow ACLS algorithms and effectively work as a team while managing patients with cardiac arrests. Our simulation is a unique contribution to medical education literature in that it describes an easily applied method to introduce residents to multiple critical care concepts, including complications of ARDS and initiation of prone CPR.

Postsimulation survey data showed that residents found this to be a valuable educational experience. Despite the various levels of learners, nearly all survey respondents felt that the content was appropriate for their level of training. Residents agreed that all of the key educational objectives of the simulation were met, and nearly all thought that the material covered would be useful for their future clinical practice.

The setup, implementation, and execution of this simulation proceeded without any major difficulties. One lesson learned after the first run of the simulation was that some residents felt a bit flustered upon entering the simulation room with alarms sounding. In order to address this, we provided the residents with more time to read the clinical scenario (Appendix B) outside of the simulation room prior to placing them in the chaotic environment of the simulated setting. The simulation could have been enhanced with the participation of nurses, pharmacists, and respiratory therapists to create a more realistic multidisciplinary code blue learning scenario. It also could have been improved by reducing the size of the resident teams involved in the simulation from an average of six to an average of three or four. Smaller teams would better simulate the size of real clinical teams and

Copyright © 2021 Sinha et al. This is an open-access publication distributed under the terms of the Creative Commons Attribution license.

give each resident a louder voice in the critical decision-making of the case.

One of the largest limitations of this simulation was our low survey response rate (41%). Following the simulation, we emailed all of the residents who had participated a link to complete the survey. However, the survey was not mandatory, and trainees did not receive the email until they had already returned to clinical duties. Therefore, though our survey results suggested that trainees found the simulation useful, it was possible that individuals who took the time to complete the survey felt differently (or more strongly) compared to those who did not. We could also have improved our survey instrument by including a unique question for each identified learning objective instead of assessing them as a group. Lastly, we could have included some type of formative assessment (or pre- and postsimulation knowledge assessment) in order to determine whether residents' knowledge regarding prone CPR improved postsimulation. Though trainees reported that the case was useful for their future practice and that they believed that learning objectives were met, we could demonstrate improved knowledge regarding CPR in the prone position by including a more objective measurement of knowledge.

Despite the limitations to the outcome measures of this case, we received very positive feedback about the educational value of this simulation. It filled a much-needed gap in training regarding how to implement CPR in the prone patient at our institution and also afforded residents the opportunity to practice organizing as a code team and identifying reasons for cardiopulmonary arrest. This simulation is a valuable addition to residency critical care curricula, especially at institutions that deal with a high incidence of ARDS and utilize prone ventilation.

Appendices

A. Simulation Case Template.docx

B. Learner Information.docx

C. Debriefing Materials.docx

D. Prone CPR Operating Procedure.docx

All appendices are peer reviewed as integral parts of the Original Publication.

Tejas Sinha, MD: Chief Resident, Department of Internal Medicine, The Ohio State University Wexner Medical Center; ORCID: https://orcid.org/0000-0002-8625-257X

Kyle Stinehart, MD: Fellow, Department of Internal Medicine, Division of Pulmonary, Critical Care, and Sleep Medicine, The Ohio State University Wexner Medical Center

Cashay Moorer, MPH: Medical Simulation Specialist, Clinical Skills Education and Assessment Center, The Ohio State University College of Medicine

Carleen Spitzer, MD: Assistant Professor, Department of Internal Medicine, Division of Pulmonary, Critical Care, and Sleep Medicine, The Ohio State University Wexner Medical Center

Disclosures
None to report.

Funding/Support
None to report.

Ethical Approval
Reported as not applicable.

References

1. ARDS Definition Task Force. Acute respiratory distress syndrome: the Berlin definition. *JAMA.* 2012;307(23):2526-2533. https://doi.org/10.1001/jama.2012.5669
2. Thompson BT, Chambers RC, Liu KD. Acute respiratory distress syndrome. *N Engl J Med.* 2017;377(6):562-572. https://doi.org/10.1056/NEJMra1608077
3. Bellani G, Laffey JG, Pham T, et al.; LUNG SAFE Investigators; ESICM Trials Group. Epidemiology, patterns of care, and mortality for patients with acute respiratory distress syndrome in intensive care units in 50 countries. *JAMA.* 2016;315(8):788-800. https://doi.org/10.1001/jama.2016.0291
4. Phua J, Badia JR, Adhikari NKJ, et al. Has mortality from acute respiratory distress syndrome decreased over time? A systematic review. *Am J Respir Crit Care Med.* 2009;179(3):220-227. https://doi.org/10.1164/rccm.200805-722OC
5. Guérin C, Reignier J, Richard JC, et al.; PROSEVA Study Group. Prone positioning in severe acute respiratory distress syndrome. *N Engl J Med.* 2013;368(23):2159-2168. https://doi.org/10.1056/NEJMoa1214103
6. Pelosi P, Tubiolo D, Mascheroni D, et al. Effects of the prone position on respiratory mechanics and gas exchange during acute lung injury. *Am J Respir Crit Care Med.* 1998;157(2):387-393. https://doi.org/10.1164/ajrccm.157.2.97-04023
7. Mure M, Martling CR, Lindahl SGE. Dramatic effect on oxygenation in patients with severe acute lung insufficiency treated in the prone position. *Crit Care Med.* 1997;25(9):1539-1544. https://doi.org/10.1097/00003246-199709000-00022

Copyright © 2021 Sinha et al. This is an open-access publication distributed under the terms of the Creative Commons Attribution license.

8. Blanch L, Mancebo J, Perez M, et al. Short-term effects of prone position in critically ill patients with acute respiratory distress syndrome. *Intensive Care Med.* 1997;23(10):1033-1039. https://doi.org/10.1007/s001340050453

9. Jolliet P, Bulpa P, Chevrolet JC. Effects of the prone position on gas exchange and hemodynamics in severe acute respiratory distress syndrome. *Crit Care Med.* 1998;26(12):1977-1985. https://doi.org/10.1097/00003246-199812000-00023

10. Berlin DA, Gulick RM, Martinez FJ. Severe Covid-19. *N Engl J Med.* Published online 2020. https://doi.org/10.1056/NEJMcp2009575

11. Ghelichkhani P, Esmaeili M. Prone position in management of COVID-19 patients; a commentary. *Arch Acad Emerg Med.* 2020;8(1):e48. https://doi.org/10.22037/aaem.v8i1.674

12. Mazer SP, Weisfeldt M, Bai D, et al. Reverse CPR: a pilot study of CPR in the prone position. *Resuscitation.* 2003;57(3):279-285. https://doi.org/10.1016/S0300-9572(03)00037-6

13. Cave DM, Gazmuri RJ, Otto CW, et al. Part 7: CPR techniques and devices: 2010 American Heart Association Guidelines for Cardiopulmonary Resuscitation and Emergency Cardiovascular Care. *Circulation.* 2010;122(18)(suppl 3):S720-S728. https://doi.org/10.1161/CIRCULATIONAHA.110.970970

14. Tofil NM, Dollar J, Zinkan L, et al. Performance of anesthesia residents during a simulated prone ventricular fibrillation arrest in an anesthetized pediatric patient. *Paediatr Anaesth.* 2014;24(9):940-944. https://doi.org/10.1111/pan.12406

15. Hegarty C, Nelson J. Shortness of breath/PEA. *MedEdPORTAL*. 2008;4:1071. https://doi.org/10.15766/mep_2374-8265.1071

16. O'Neill J, Docherty M, Fitch M. Cystic fibrosis: respiratory failure with pneumothorax. *MedEdPORTAL*. 2014;10:9851. https://doi.org/10.15766/mep_2374-8265.9851

17. Rubin A, Ten Eyck R. Gunshot wound simulation case. *MedEdPORTAL*. 2012;8:9084. https://doi.org/10.15766/mep_2374-8265.9084

18. Beck J, Romo C, Denmark K. Pharmacology simulation scenario: tension pneumothorax. *MedEdPORTAL.* 2010;6:7966. https://doi.org/10.15766/mep_2374-8265.7966

19. Ten Eyck R. Status asthmaticus with development of tension pneumothorax post-intubation. *MedEdPORTAL.* 2008;4:787. https://doi.org/10.15766/mep_2374-8265.787

20. Zhang XC, Golden A, Lindquist D. Troubleshooting postintubation hypoxia: a simulation case for emergency medicine residents. *MedEdPORTAL*. 2016;12:10397. https://doi.org/10.15766/mep_2374-8265.10397

21. Cooper AZ. Standard operating procedure: CPR in prone patients. Presented at: Ohio State Wexner Medical Center Code Blue and Emergency Response Team/Critical Care Quarterly Meeting Consortium; February 20, 2019; Columbus, OH.

22. Barker J, Koeckerling D, West R. A need for prone position CPR guidance for intubated and non-intubated patients during the COVID-19 pandemic. *Resuscitation*. 2020;151:135-136. https://doi.org/10.1016/j.resuscitation.2020.04.029

23. Douma MJ, MacKenzie E, Loch T, et al. Prone cardiopulmonary resuscitation: a scoping and expanded grey literature review for the COVID-19 pandemic. *Resuscitation.* 2020;155:103-111. https://doi.org/10.1016/j.resuscitation.2020.07.010

24. Ludwin K, Szarpak L, Ruetzler K, et al. Cardiopulmonary resuscitation in the prone position: a good option for patients with COVID-19. *Anesth Analg.* 2020;131(3):e172-e173. https://doi.org/10.1213/ANE.0000000000005049

Received: March 25, 2020
Accepted: October 11, 2020
Published: February 11, 2021

Copyright © 2021 Sinha et al. This is an open-access publication distributed under the terms of the Creative Commons Attribution license.
